# Supplementary material for: Structural insights into Wnt/β-catenin signaling regulation by LGR4, R-spondin, and ZNRF3
Source: Nat Commun. 2025 Oct 1;16:8337. doi: 10.1038/s41467-025-64129-z (PMC12488874; doi:10.1038/s41467-025-64129-z)
Supplement: Supplementary file 1 — Supplementary Information [file 41467_2025_64129_MOESM1_ESM.pdf]

```
                                                            29
                                                            LRRNT
LGR4        ------MPGPLGLLCFLALGLLGSAGPSGAAPPLCAAPCSCDGDR----RVDCSGKGLTA   50
LGR5        -MDTSRLGVLLSLPVLLQLATGGSSPRSGVLLRGCPTHCHCEPDGRMLLRVDCSDLGLSE   59
LGR6        MPSPPGLRALWLCAALCASRRAGGAPQPGPGPTACPAPCHCQEDG-IMLSADCSELGLSA   59

                   57   60  LRR1        79   82    LRR2           103 106
LGR4        VPEGLSAFTQALDISMNNITQLPEDAFKNFPFLEELQLAGNDLSFIHPKALSGLKELKVL  110
LGR5        LPSNLSVFTSYLDLSMNNISQLLPNPLPSLRFLEELRLAGNALTYIPKGAFTGLYSLKVL  119
LGR6        VPGDLDPLTAYLDLSMNNLTELQPGLFHHLRFLEELRLSGNHLSHIPGQAFSGLYSLKIL  119

                 LRR3       127  130     LRR4          151  154     LRR5
LGR4        TLQNNQLKTVPSEAIRGLSALQSLRLDANHITSVPEDSFEGLVQLRHLWLDDNSLTEVPV  170
LGR5        MLQNNQLRHVPTEALQNLRSLQSLRLDANHISYVPPSCFSGLHSLRHLWLDDNALTEIPV  179
LGR6        MLQNNQLGGIPAEALWELPSLQSLRLDANLISLVPERSFEGLSSLRHLWLDDNALTEIPV  179

                  177178     LRR6        199  202     LRR7          223 226
LGR4        HPLSNLPTLQALTLALNKISSIPDFAFTNLSSLVVLHLHNNKIRSLSQHCFDGLDNLETL  230
LGR5        QAFRSLSALQAMTLALNKIHHIPDYAFGNLSSLVVLHLHNNRIHSLGKKCFDGLHSLETL  239
LGR6        RALNNLPALQAMTLALNRISHIPDYAFQNLTSLVVLHLHNNRIQHLGTHSFEGLHNLETL  239

                 LRR8       247  249     LRR9          270  273    LRR10
LGR4        DLNYNNLGEFPQAIKALPSLKELGFHSNSISVIPDGAFDGNPLLRTIHLYDNPLSFVGNS  290
LGR5        DLNYNNLDEFPTAIRTLSNLKELGFHSNNIRSIPEKAFVGNPSLITIHFYDNPIQFVGRS  299
LGR6        DLNYNKLQEFPVAIRTLGRLQELGFHNNNIKAIPEKAFMGNPLLQTIHFYDNPIQFVGRS  299

                294  297    LRR11        317  320    LRR12         341 344
LGR4        AFHNLSDLHSLVIRGASMVQQFPNLTGTVHLESLTLTGTKISSIPNNLCQEQKMLRTLDL  350
LGR5        AFQHLPELRTLTLNGASQITEFPDLTGTANLESLTLTGAQISSLPQTVCNQLPNLQVLDL  359
LGR6        AFQYLPKLHTLSLNGAMDIQEFPDLKGTTSLEILTLTRAGIRLLPSGMCQQLPRLRVLEL  359

                 LRR13       365 366     LRR14         387  390    LRR15
LGR4        SYNNIRDLPSFNGCHALEEISLQRNQIYQIKEGTFQGLISLRILDLSRNLIHEIHSRAFA  410
LGR5        SYNLLEDLPSFSVCQKLQKIDLRHNEIYEIKVDTFQQLLSLRSLNLAWNKIAIIHPNAFS  419
LGR6        SHNQIEELPSLHRCQKLEEIGLQHNRIWEIGADTFSQLSSLQALDLSWNAIRSIHPEAFS  419

            411  414     LRR16       432  435     LRR17         455456
LGR4        TLGPITNLDVSFNELTSFPTEGLNGLNQLKLVGNFKLKEALAAKDFVNLRSLSVPYAYQC  470
LGR5        TLPSLIKLDLSSNLLSSFPITGLHGLTHLKLTGNHALQSLISSENFPELKVIEMPYAYQC  479
LGR6        TLHSLVKLDLTDNQLTTLPLAGLGGLMHLKLKGNLALSQAFSKDSFPKLRILEVPYAYQC  479

                                         LRRCT
LGR4        CAFWGCDSYANLNTEDNSLQDHSVAQEKGTADAANVTSTLE--------------NEEHS  516
LGR5        CAFGVCENAYKISNQWNKGDNSSMDDLHKKDAGMFQAQDER----DLEDFLLDFEEDLKA  535
LGR6        CPYGMCASFFKASGQWEAEDLHLDDEESSKRPLGLLARQAENHYDQDLDELQLEMEDSKP  539

                    525
LGR4        QIIIHCTPS   525
LGR5        LHSVQCSPS   544
LGR6        HPSVQCSPT   548
```

Residues of LGR4-RSPO2 interface

Residues of LGR4-ZNRF3* interface

Supplementary Fig. 1 | Sequence alignment of LGR4-6 extracellular region.
The sequence alignment of the extracellular regions of human LGR4, LGR5, and LGR6 was calculated using Clustal Omega. Residues are colored to indicate the degree of similarity; red residues display the highest similarity, followed by green, blue, and black (lowest similarity). The residues involved in RSPO2 binding are highlighted in yellow. The residues involved in the LGR4-ZNRF3* interface in the LGR4-RSPO2-ZNRF3$_{2:2:2}$ complex are highlighted in green.

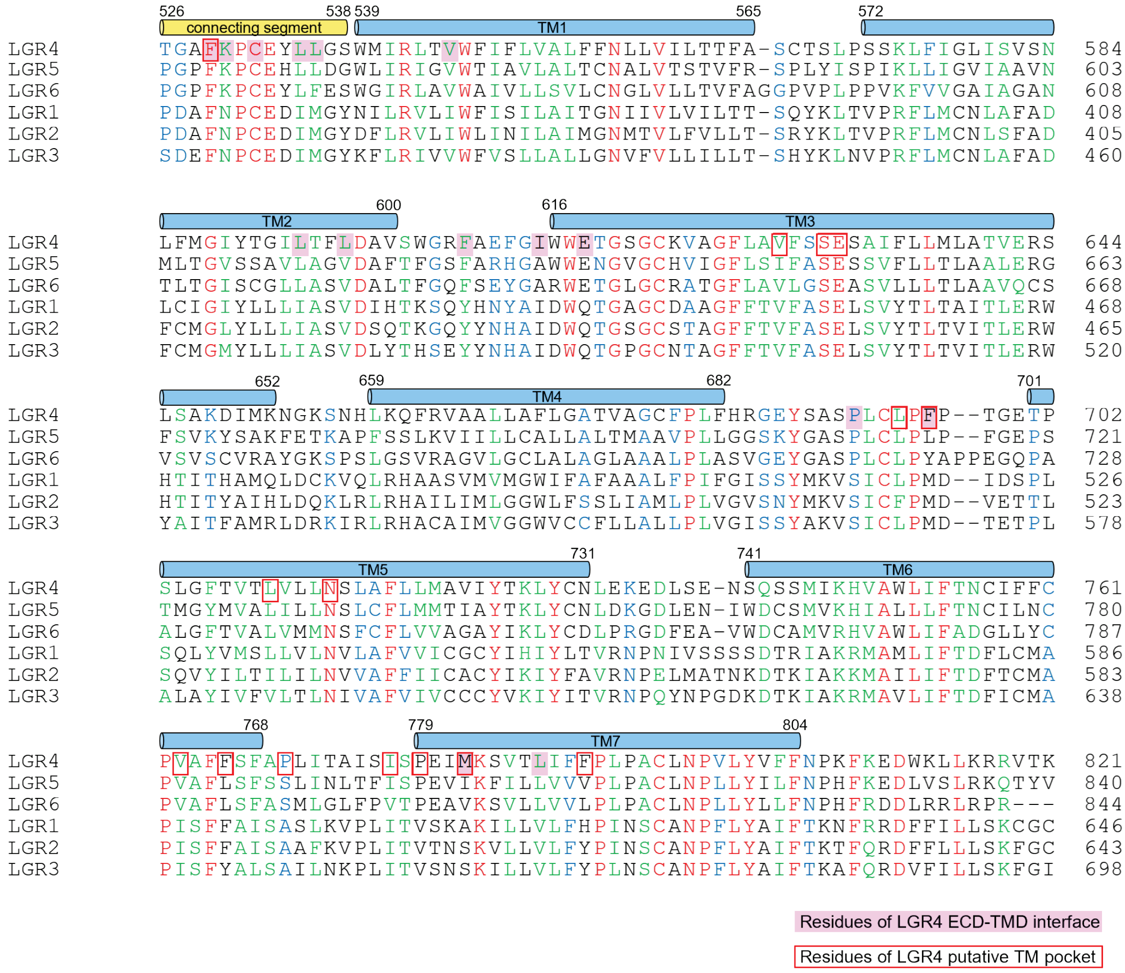

Supplementary Fig. 2 | Sequence alignment of LGR1-6 transmembrane region.

The sequence alignment of the connecting segment and transmembrane region of human LGR1-6 was calculated using Clustal Omega. Residues are colored to indicate the degree of similarity; red residues display the highest similarity, followed by green, blue, and black (lowest similarity). The residues in the LGR4 ECD-TMD interface are highlighted in pink. The residues that form the putative transmembrane pocket of LGR4 are highlighted in red boxes.

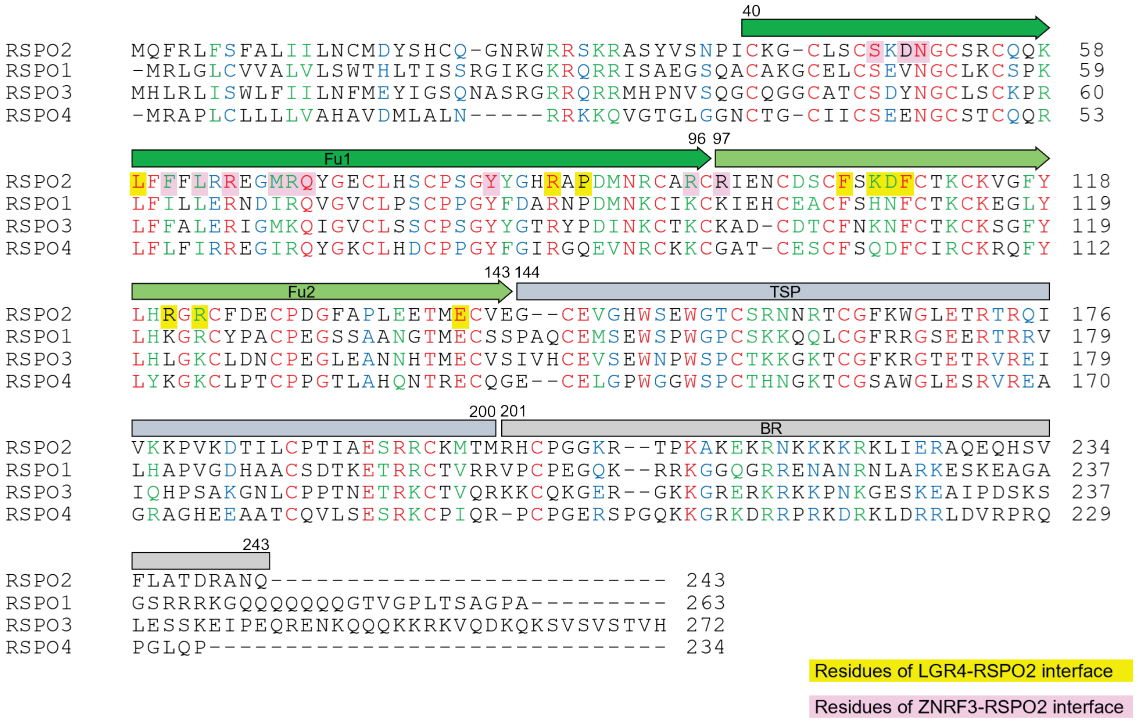

Supplementary Fig. 3 | Sequence alignment of RSPO1-4.
The sequence alignment of human RSPO1-4 was calculated using the Clustal Omega. The residues are colored to indicate the degree of similarity; red residues display the highest similarity, followed by green, blue, and black (lowest similarity). The residues involved in LGR4 binding are highlighted in yellow. The residues involved in ZNRF3 binding are highlighted in pink.

```
                                                                56
ZNRF3     MRPRSGGRPGATGRRRRRLRRRPRGLRCSRLPPPPPLPLLLGLLLAAAGPGAARAKETAF   60
RNF43     ----------MSGGHQLQLAALWPWLLMATLQAG---FGRTGLVLAAAVESERSAEQKAI   47

                                  ECD
ZNRF3     VEVVLFESSPSGDYTTYTTGLTGRFSRAGATLSAEGEIVQMHPLGLCNNNDEEDLYEYGW  120
RNF43     IRVIPLKMDPTG---KLNLTLEGVFAGVAEITPAEGKLMQSHPLYLCNASDD-DNLEPGF  102

ZNRF3     VGVVKLEQPELDPKPCLTVLGKAKRAVQRGATAVIFDVSENPEAIDQLNQGSEDPLKRPV  180
RNF43     ISIVKLESPRRAPRPCLSLASKARMAGERGASAVLFDITEDRAAAEQLQQPLG--LTWPV  161

                                      207 208
                                             TMD
ZNRF3     VYVKGADAIKLMNIVNKQKVARARIQHRPPRQPTEYFDMGIFLAFFVVVSLVCLILLVKI  240
RNF43     VLIWGNDAEKLMEFVYKNQKAHVRIELK---EPPAWPDYDVWILMTVVGTIFVIILASVL  218

                                      267                         292
ZNRF3     KLKQRRSQNSMNRLAVQALEKMETRKFNSKSKGRREGSCGALDTLSSSSTS-DCAICLEK  299
RNF43     RIRCRPRHSRPDPLQQRTAWAISQLATRRYQASCRQARGEWPDSGSSCSSAPVCAICLEE  278

                                                     338
                       RING
ZNRF3     YIDGEELRVIPCTHRFHRKCVDPWLLQHHTCPHCRHNIIEQKGNPSAVCVETSNLSRGRQ  359
RNF43     FSEGQELRVISCLHEFHRNCVDPWLHQHRTCPLCMFNITEGDSFSQSLGPSRSYQEPGRR  338

ZNRF3     QRVTLPVHYPGRVHRTNAIPAYPTRTSMDSHGNPVTLLTMDRHGEQSLYSPQTPAYIRSY  419
RNF43     LHLIRQHPGHAHYHLPAAYLLGPSRSAVARPPRPGPFLPSQEPG----------------  336

ZNRF3     PPLHLDHSLAAHRCGLEHRAYSPAHPFRRPKLSGRSFSKAACFSQYETMYQHYYFQGLSY  479
RNF43     ---------MGPRHHRFPRAAHPRAPGEQQRLAG---------------AQHPYAQGWGL  418

ZNRF3     PEQEGQSPPSLAPRGPARAFPPSGSGSLLFPTVVHVAPPSHLESGSTSSFSCYHGHRSVC  539
RNF43     SHLQSTSQHPAACPVPLRRARPPDS------------------SGSGESYCTER------  454

ZNRF3     SGYLADCPGSDSSSSSSSGQCHCSSSDSVVDCTEVSNQGVYGSCSTFRSSLSSDYDPFIY  599
RNF43     SGYLADGPASDS----SSGPCHGSSSDSVVNCTDISLQGVHGSSSTFCSSLSSDFDPLVY  510

ZNRF3     RSRSPCRASEAGGSGSSGRGPALCFEGSPPPEELPAVHSHGAGRGEPWPGPASPSGDQVS  659
RNF43     CSPKGDPQRVDMQPSVTSRPRSLDS--------------------------VVPTGETQV  544

ZNRF3     TCSLEMNYSSNSSLEHRGPNSSTSEVGLEASPGAAPDLRRTWKGGHELPSCACCCEPQPS  719
RNF43     SSHVHYHRHRHHHYKKRFQWHGRKPGPETGVPQSRPPIPRTQ--------------PQPE  590

ZNRF3     PAGPSAGAAGSSTLFLGPHLYEGSGPAGGEPQSGSSQGLYGLHPDHLPRTDGVKYEGLPC  779
RNF43     PPSPDQQVTRSNSAAPSGRLSNPQCPRALPEPAPGPVDASSICPSTSSLFN------LQK  644

ZNRF3     CFYEEKQVARGGGGGSGCYTEDYSVSVQYTLTEEPPPGCYPGARDLSQRIPIIPEDVDCD  839
RNF43     SSLSARHPQRKRRGGP----------------SEPTPGSRPQDATVHPACQIFPHYTPSV  688

ZNRF3     LGLPSDCQGTHSLGSWGGTRGPDTPRPHRGLGATREEERALCCQARALLRPGCPPEEAGA  899
RNF43     AYPWSPEAHPLICGPPGLDKRLLPETPGP--CYSNSQPVWLCLTPRQPLEPHPPGEGPSE  746

ZNRF3     VRANFPSALQDTQESSTTATEAAGPRSHSADSSSPGA  936
RNF43     WSSDTAEGRPCPYPHCQVLSAQPGSEEELEELCEQAV  783
```

Residues of RSPO2-ZNRF3 interface

Residues of LGR4-ZNRF3* interface

Residues of ZNRF3-ZNRF3* interface

Supplementary Fig. 4 | Sequence alignment of ZNRF3 and RNF43.

The sequence alignment of human ZNRF3 and RNF43 was calculated using Clustal Omega. The residues are colored to indicate the degree of similarity; red residues display the highest similarity, followed by green, blue, and black (lowest similarity). The residues involved in RSPO2 binding are highlighted in yellow. The residues at the LGR4-ZNRF3* interface in the LGR4-RSPO2-ZNRF3$_{2:2:2}$ complex are highlighted in blue. The residues involved in the ZNRF3 ECD dimerization interface are highlighted in red boxes.

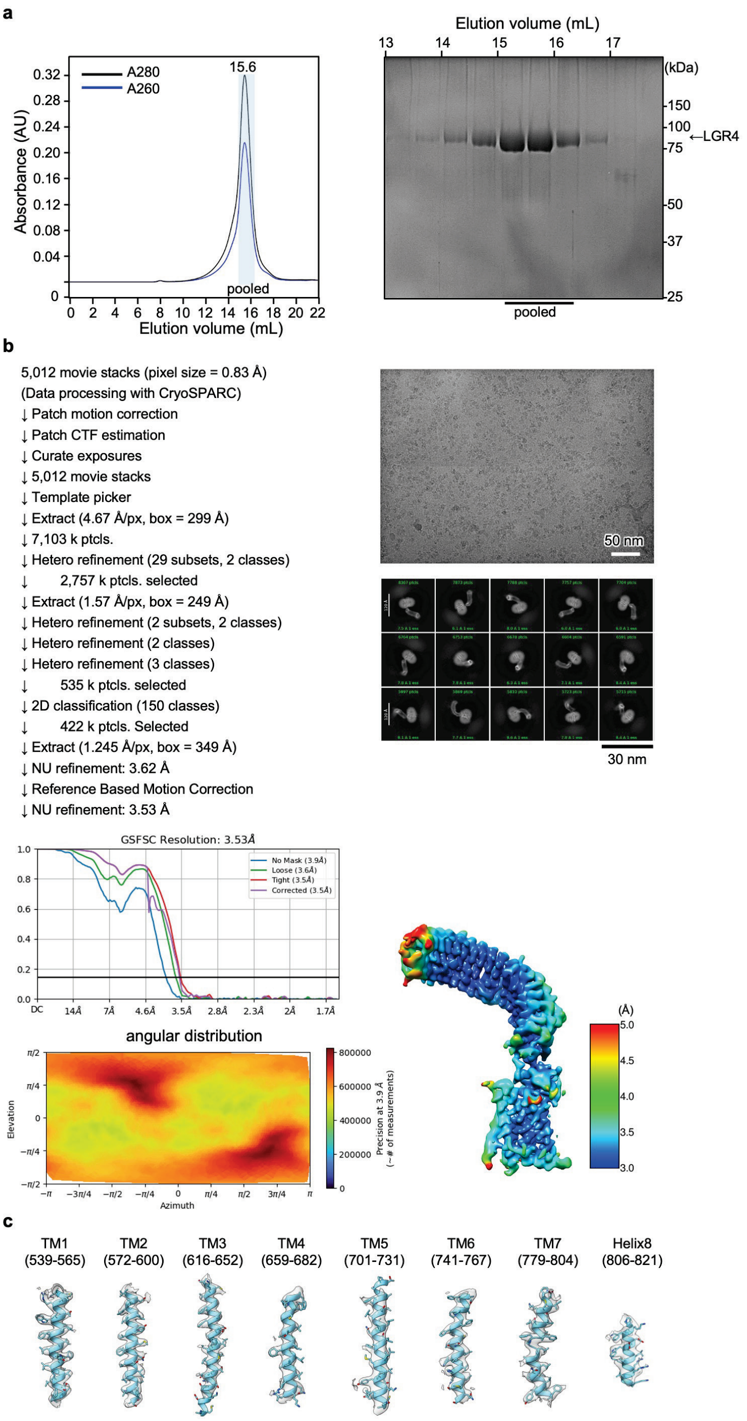

Supplementary Fig. 5 | Sample preparation and Cryo-EM analysis of LGR4.

a, Size exclusion chromatography profile of LGR4 purified from Expisf9 cells. Absorbance profiles (left) and Sodium Dodecyl Sulfate-Polyacrylamide Gel Electrophoresis (SDS-PAGE) analysis of the peak fractions stained with Coomassie Brilliant Blue (CBB, right) are shown. Pooled fractions are indicated by bars. Absorbances at 280 nm and 260 nm are shown as black and blue lines, respectively. Source data are provided as a Source Data file.

b, Cryo-EM data processing of LGR4. The motion-corrected micrograph, two-dimensional (2D) class averages, and gold-standard Fourier shell correlation (FSC) curves of the final three-dimensional (3D reconstruction (resolution cutoff at FSC = 0.143), and final 3D map (colored according to local resolution) are shown. The 2D class averages were calculated using the refined particles for the final reconstruction.

c, Cryo-EM density maps of LGR4 showing the density of 7-TM helices of LGR4. The map thresholds were adjusted as appropriate for each region due to differences in local resolution.

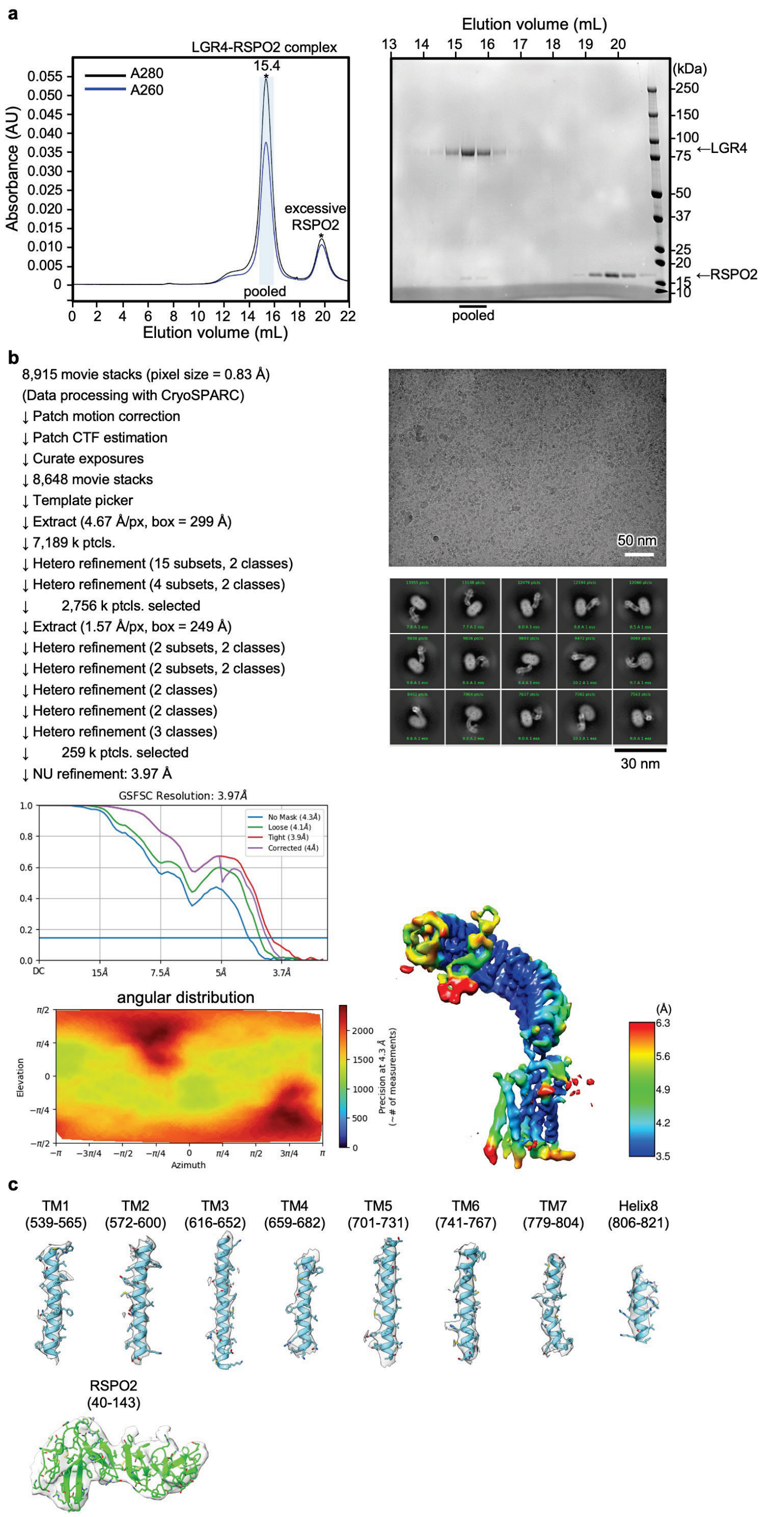

Supplementary Fig. 6 | Sample preparation and Cryo-EM analysis of the LGR4-RSPO2 complex.

a, SEC profile of the LGR4-RSPO2 complex. Absorbance profiles (left) and SDS-PAGE analysis of the peak fractions stained with CBB (right) are shown. The pooled fractions are indicated by bars. Absorbances at 280 nm and 260 nm are shown as black and blue lines, respectively. Source data are provided as a Source Data file.

b, Cryo-EM data processing of the LGR4-RSPO2 complex. The motion-corrected micrograph, 2D class averages, and gold-standard FSC curves of the final 3D reconstruction (resolution cutoff at FSC = 0.143), and final 3D map (colored according to local resolution) are shown. The 2D class averages were calculated using the refined particles for the final reconstruction.

c, Cryo-EM density maps of the LGR4-RSPO2 complex showing the densities of LGR4 7-TM helices and RSPO2.

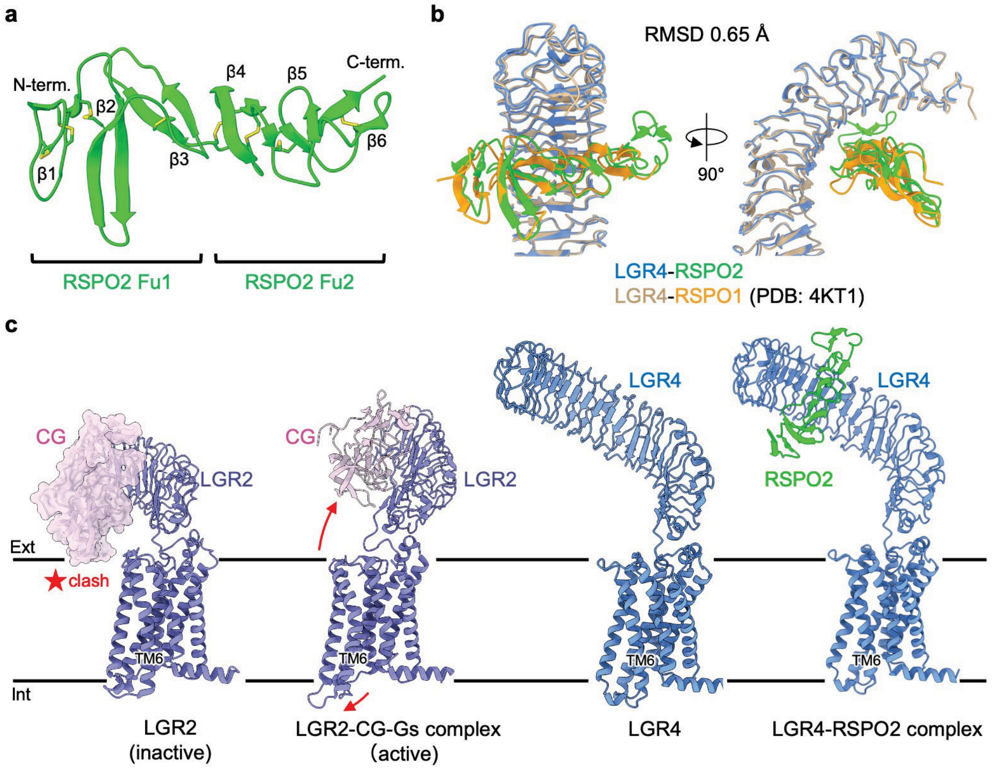

Supplementary Fig. 7 | The LGR4-RSPO2 complex and related structures.

a, The structure of RSPO2 Fu1-2 domains (this study), consisting of 6 pairs of β-hairpins stabilized by disulfide bonds (yellow).

b, Superimposition of the cryo-EM structure of the LGR4-RSPO2 complex (this study) on the crystal structure of the LGR4-RSPO1 complex (PDB: 4KT1).

c, Comparison between LGR2 and LGR4 upon binding to their respective ligands. The structures include the inactive LGR2 (PDB: 7FIJ) with modeled chorionic gonadotropin (CG) (left), active LGR2-CG-Gs complex (PDB: 7FII) (middle left), LGR4 (this study) (middle right), and the LGR4-RSPO2 complex (this study) (right). In LGR2, the potential steric clash of the CG with the membrane resulted in the upward rotation of the ECD and outward movement of TM6. In contrast, LGR4 does not undergo these conformational changes upon RSPO2 binding.

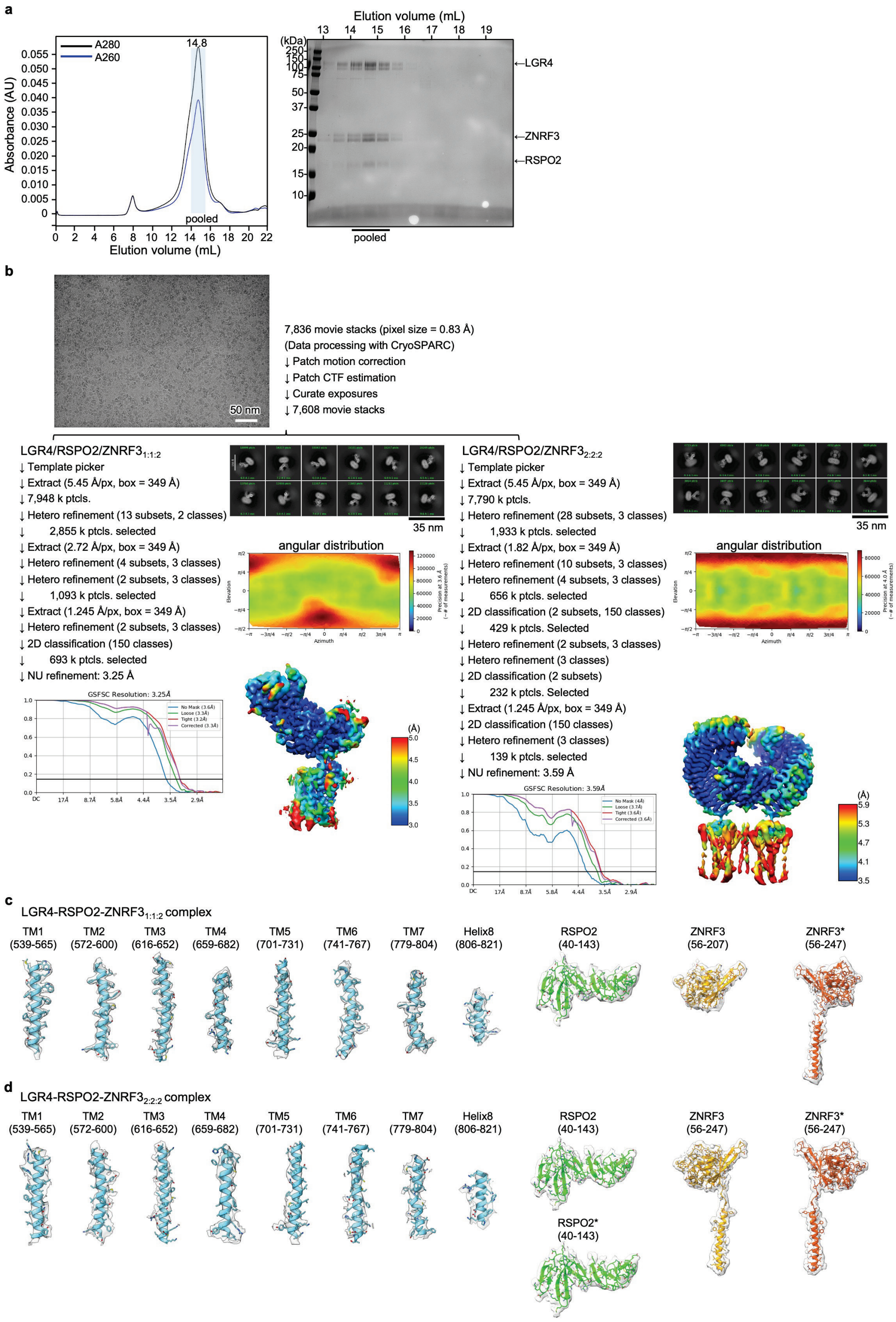

Supplementary Fig. 8 | Sample preparation and Cryo-EM analysis of the LGR4-RSPO2-ZNRF3 complex.

a, SEC profile of the LGR4-RSPO2-ZNRF3 complex. Absorbance profiles (left) and SDS-PAGE analysis of the peak fractions stained with CBB (right) are shown. The pooled fractions are indicated by bars. Absorbances at 280 nm and 260 nm are shown as black and blue lines, respectively. Source data are provided as a Source Data file.

b, Cryo-EM data processing of the LGR4-RSPO2-ZNRF3 complex. The motion-corrected micrograph, 2D class averages, gold-standard FSC curves of the final 3D reconstruction (resolution cutoff at FSC = 0.143), and final 3D map (colored according to the local resolution) are shown. The 2D class averages were calculated using the refined particles for the final reconstruction.

c, d, Cryo-EM density maps of the LGR4-RSPO2-ZNRF3$_{1:1:2}$ complex (**c**) and the LGR4-RSPO2-ZNRF3$_{2:2:2}$ complex (**d**) showing the density of LGR4 7-TM helices, RSPO2, and ZNRF3.

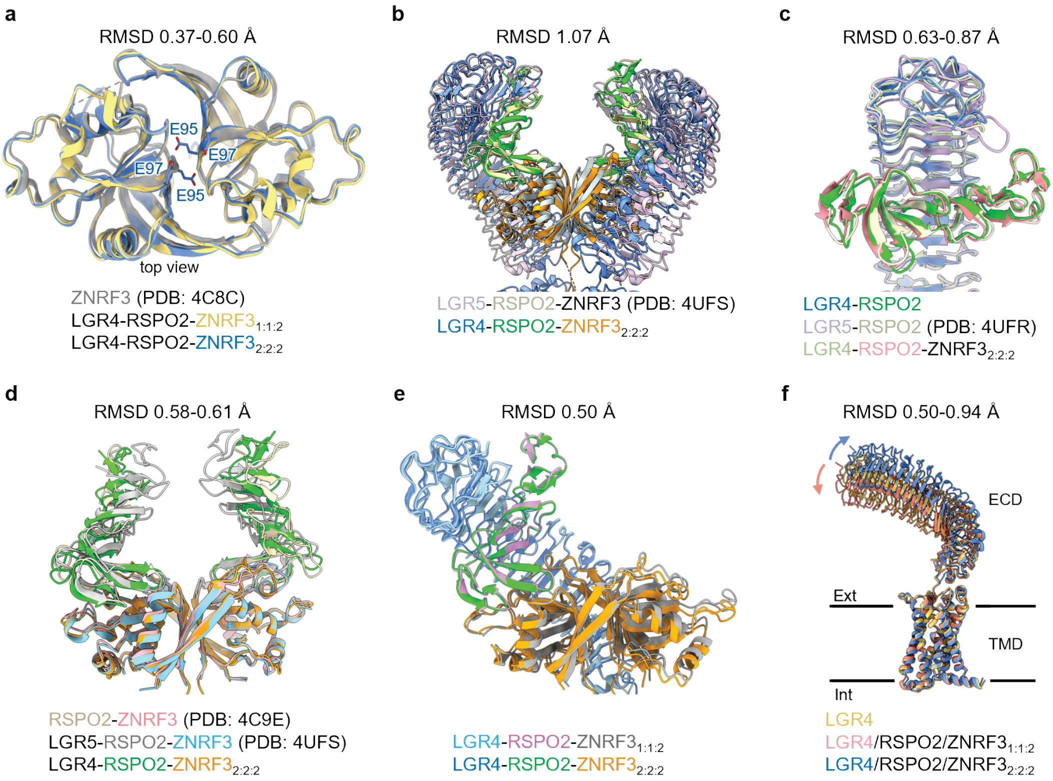

Supplementary Fig. 9 | Structural comparisons of the LGR4-RSPO2-ZNRF3 complex with previous studies.

a, Superimposition of the cryo-EM structure of ZNRF3 dimers from the LGR4-RSPO2-ZNRF3 complex (this study) with the crystal structure of the ZNRF3 ECD dimer (PDB: 4C8C).

b, Superimposition of the cryo-EM structure of the LGR4-RSPO2-ZNRF$_{2:2:2}$ complex (this study) with that of the LGR5-RSPO2-ZNRF3 complex (PDB: 4UFS).

c, Superimposition of the cryo-EM structure of the LGR4-RSPO2 complex (this study), the LGR4-RSPO2-ZNRF$_{2:2:2}$ complex (this study) with the crystal structure of the LGR5-RSPO2 complex (PDB: 4UFR).

d, Superimposition of the cryo-EM structure of the LGR4-RSPO2-ZNRF$_{2:2:2}$ complex (this study) with the crystal structures of the RSPO2-ZNRF3 complex (PDB: 4C9E) and the LGR5-RSPO2-ZNRF3 complex (PDB: 4UFS), with LGR4/5 are not shown.

e, Superimposition of the cryo-EM structures of the LGR4-RSPO2-ZNRF$_{1:1:2}$ and LGR4-RSPO2-ZNRF$_{2:2:2}$ complexes (this study), focusing on the ECD of LGR4-RSPO2-ZNRF$_{1:1:2}$.

f, Superimposition of the cryo-EM structures of LGR4 in the apo state, the LGR4-RSPO2-ZNRF$_{1:1:2}$ and LGR4-RSPO2-ZNRF$_{2:2:2}$ complexes (this study), focusing on LGR4.

RMSD: Root Mean Square Deviation.

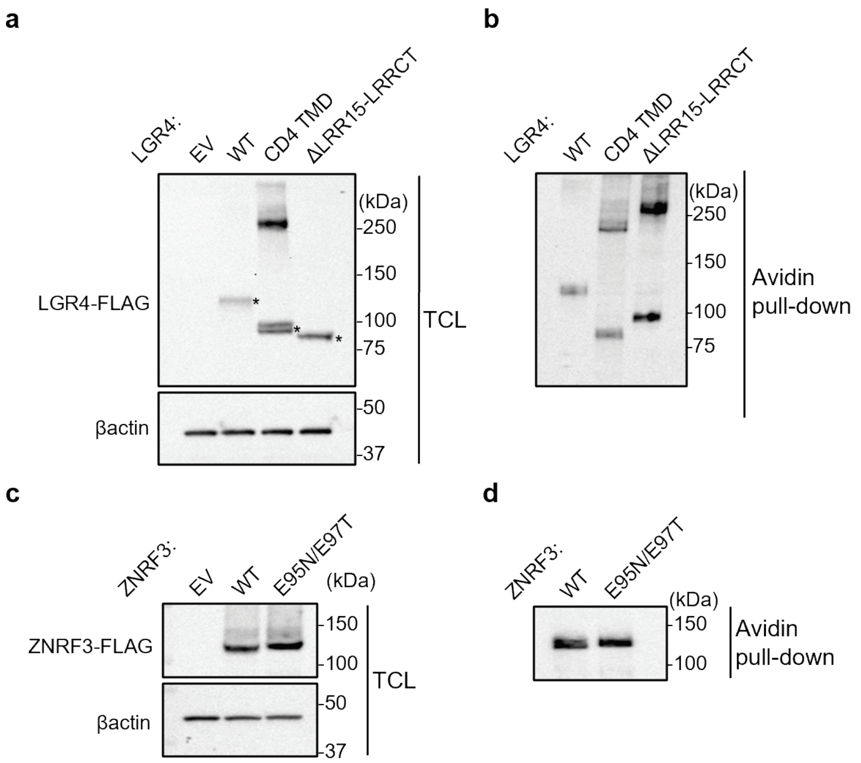

Supplementary Fig. 10 | Expression and cell surface localization of LGR4 and ZNRF3

a, Western blot analysis of full-length wild-type (WT) LGR4, LGR4 (CD4 TMD) mutant, or LGR4 (ΔLRR15-LRRCT) mutant. LGR4 and LGR5 knockdown HEK293T cells were transfected with empty vector (EV) or FLAG-tagged LGR4 expression plasmids as in TOPFlash reporter assay, and the total cell lysates (TCL) were analyzed in western blotting with anti-FLAG or anti-βactin antibodies.

b, Western blot analysis of cell surface LGR4 (WT), LGR4 (CD4 TMD), or LGR4 (ΔLRR15-LRRCT). HEK293T cells were transfected with FLAG-tagged LGR4 expression plasmids and subjected to membrane biotinylation, and biotinylated membrane proteins were isolated with avidin beads and analyzed by western blotting with anti-FLAG antibodies.

c, Western blotting analysis of full-length that ZNRF3 (WT) or ZNRF3 (E95N/E97T). ZNRF3 and RNF43 knockdown HEK293T cells were transfected with empty vector (EV) or FLAG-tagged ZNRF3 expression plasmids, and the total cell lysates were analyzed as in **a**.

d, Western blot analysis of cell surface ZNRF3 (WT) or ZNRF3 (E95N/E97T). HEK293T cells were transfected with FLAG-tagged ZNRF3 expression plasmids, treated with 10 μM MG132 for 6 h to prevent proteasomal degradation, and subjected to membrane biotinylation. The biotinylated membrane proteins were isolated with avidin beads and analyzed by western blotting with anti-FLAG antibodies.

These images are representative of three independent experiments. Source data are provided as a Source Data file.

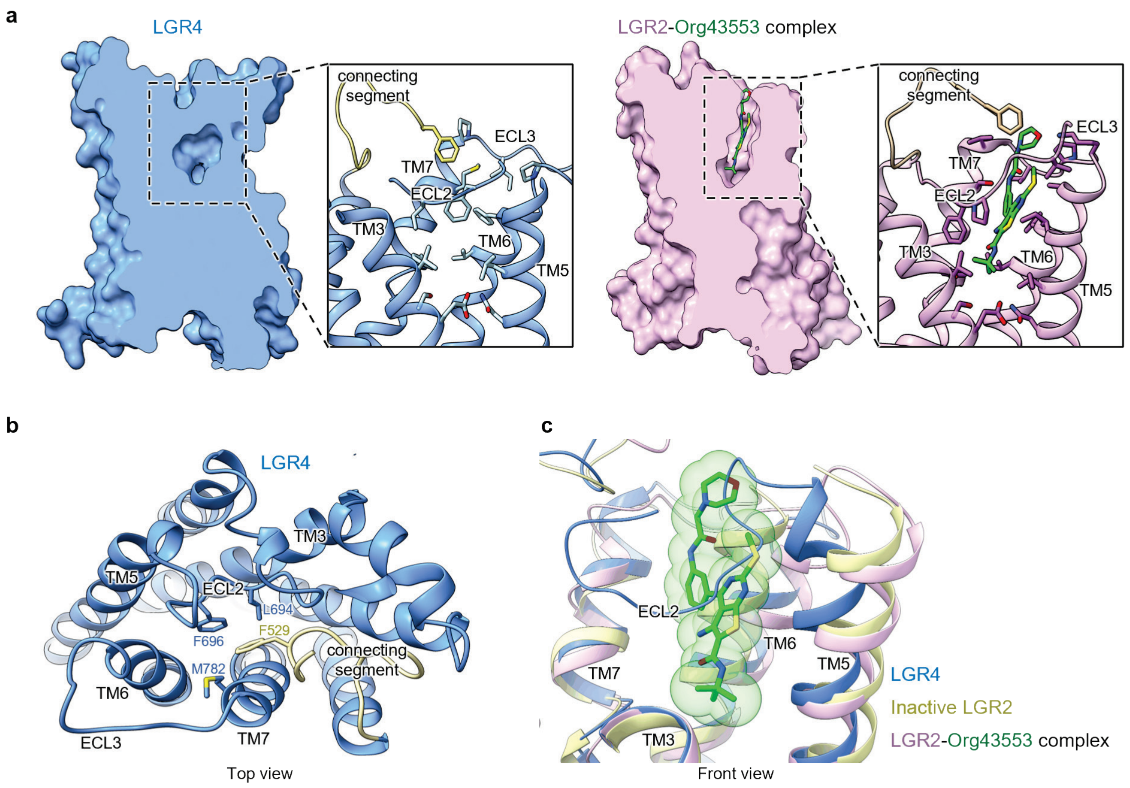

Supplementary Fig. 11 | Structure of the putative transmembrane pocket of LGR4

a, Structural comparison of the putative transmembrane pocket of LGR4 (left, blue) (this study) with that of LGR2 bound to the allosteric agonist Org43553 (right, pink; PDB: 4FII).

b, The top view of the putative transmembrane pocket of LGR4.

c, Structural comparison of the transmembrane pockets of LGR4 in the apo-state (this study), the inactive LGR2 (PDB: 7FIJ), and the active LGR2-Org43553 complex (PDB: 7FII).

Supplementary Table 1 | Cryo-EM data collection, refinement, and validation statistics

**Data**	LGR4	LGR4-RSPO2	LGR4-RSPO2-ZNRF3$_{1:1:2}$	LGR4-RSPO2-ZNRF3$_{2:2:2}$
**EMDB ID**	EMD-62218	EMD-62219	EMD-62220	EMD-62221
**PDB ID**	9KB6	9KB7	9KB8	9KB9
**Data collection and processing**				
Magnification	105,000	105,000	105,000	105,000
Voltage (kV)	300	300	300	300
Electron exposure ($e^{-}/Å^2$)	61	48	48	48
Defocus range (μm)	-0.8 to -1.6	-1.0 to -2.0	-1.0 to -2.0	-0.8 to -2.0
Pixel size (Å)	0.83	0.83	0.83	0.83
Symmetry imposed	*C*1	*C*1	*C*1	*C*2
Initial particle images (no.)	7,103,491	11,352,830	9,781,000	217,604
Final particle images (no.)	421,944	258,708	693,808	139,067
Map resolution (Å)	3.5	4.0	3.3	3.6
FSC threshold	0.143	0.143	0.143	0.143
**Refinement**				
Model resolution (Å)	3.5	4.0	3.3	3.6
FSC threshold	0.5	0.5	0.5	0.5
Map sharpening *B* factor ($Å^2$)	141.1	152.5	129.4	117.0
Model composition				
Non-hydrogen atoms	5,922	6,748	9,396	16,352
Protein residues	752	856	1,197	2,084
B factors ($Å^2$)	162.10	183.40	149.60	190.89
R.m.s. deviations				
Bond lengths (Å)	0.005	0.004	0.003	0.006
Bond angles (°)	1.024	1.053	0.607	1.036
**Validation**				
MolProbity score	1.63	1.88	1.68	2.26
Clashscore	6.14	14.55	5.96	10.63
Poor rotamers (%)	0.91	0.66	1.63	2.63
Ramachandran plot				
Favored (%)	95.72	96.71	96.88	94.15
Allowed (%)	3.88	2.93	3.12	5.75
Disallowed (%)	0.40	0.35	0.00	0.10
