# Supplementary material for: Structural insights into Wnt/β-catenin signaling regulation by LGR4, R-spondin, and ZNRF3
Source: Nat Commun. 2025 Oct 1;16:8337. doi: 10.1038/s41467-025-64129-z (PMC12488874; doi:10.1038/s41467-025-64129-z)
Supplement: Supplementary file 2 — Reporting Summary [file 41467_2025_64129_MOESM2_ESM.pdf]

nature portfolio

Corresponding author(s): Umeharu Ohto

Last updated by author(s): Aug 28, 2025

Reporting Summary

Nature Portfolio wishes to improve the reproducibility of the work that we publish. This form provides structure for consistency and transparency in reporting. For further information on Nature Portfolio policies, see our Editorial Policies and the Editorial Policy Checklist.

Statistics

For all statistical analyses, confirm that the following items are present in the figure legend, table legend, main text, or Methods section.

n/a	Confirmed	
☐	☒	The exact sample size (*n*) for each experimental group/condition, given as a discrete number and unit of measurement
☐	☒	A statement on whether measurements were taken from distinct samples or whether the same sample was measured repeatedly
☐	☒	The statistical test(s) used AND whether they are one- or two-sided<br>*Only common tests should be described solely by name; describe more complex techniques in the Methods section.*
☒	☐	A description of all covariates tested
☒	☐	A description of any assumptions or corrections, such as tests of normality and adjustment for multiple comparisons
☐	☒	A full description of the statistical parameters including central tendency (e.g. means) or other basic estimates (e.g. regression coefficient) AND variation (e.g. standard deviation) or associated estimates of uncertainty (e.g. confidence intervals)
☐	☒	For null hypothesis testing, the test statistic (e.g. *F*, *t*, *r*) with confidence intervals, effect sizes, degrees of freedom and *P* value noted<br>*Give P values as exact values whenever suitable.*
☒	☐	For Bayesian analysis, information on the choice of priors and Markov chain Monte Carlo settings
☒	☐	For hierarchical and complex designs, identification of the appropriate level for tests and full reporting of outcomes
☒	☐	Estimates of effect sizes (e.g. Cohen's *d*, Pearson's *r*), indicating how they were calculated

Our web collection on statistics for biologists contains articles on many of the points above.

Software and code

Policy information about availability of computer code

Data collection	Cryo-EM data: EPU software; protein gel or western blotting image: iBright Imaging Systems software (1.2.5); Luciferase activity: GloMax Discover (3.2.3)
Data analysis	Cryo-EM data: Chimera (1.13.1), ChimeraX (0.91), COOT (0.8.9.2), PHENIX (1.19.2-4158), cryoSPARC (v3.2.0-4.1.1); Statistics: GraphPad Prism (10.2.3.)

For manuscripts utilizing custom algorithms or software that are central to the research but not yet described in published literature, software must be made available to editors and reviewers. We strongly encourage code deposition in a community repository (e.g. GitHub). See the Nature Portfolio guidelines for submitting code & software for further information.

Data

Policy information about availability of data

All manuscripts must include a data availability statement. This statement should provide the following information, where applicable:

- Accession codes, unique identifiers, or web links for publicly available datasets
- A description of any restrictions on data availability
- For clinical datasets or third party data, please ensure that the statement adheres to our policy

The Cryo-EM maps and related structure coordinates of the LGR4, LGR4-RSPO2, LGR4-RSPO2-ZNRF31:1:2, and LGR4-RSPO2-ZNRF32:2:2 complexes have been deposited in the EMDB and PDB under accession codes EMD-62218 (PDB 9KB6), EMD-62219 (PDB 9KB7), EMD-62220 (PDB 9KB8), and EMD-62221 (PDB 9KB9), respectively. Previously determined structure coordinates used in this study are PDB 4C8C, 4UFS, 4UFR, 4C9E, 4FII, 7FIJ and 7FII. Source data are provided with this

paper.

Research involving human participants, their data, or biological material

Policy information about studies with human participants or human data. See also policy information about sex, gender (identity/presentation), and sexual orientation and race, ethnicity and racism.

Reporting on sex and gender	N/A
Reporting on race, ethnicity, or other socially relevant groupings	N/A
Population characteristics	N/A
Recruitment	N/A
Ethics oversight	N/A

Note that full information on the approval of the study protocol must also be provided in the manuscript.

Field-specific reporting

Please select the one below that is the best fit for your research. If you are not sure, read the appropriate sections before making your selection.

- [x] Life sciences
- [ ] Behavioural & social sciences
- [ ] Ecological, evolutionary & environmental sciences

For a reference copy of the document with all sections, see nature.com/documents/nr-reporting-summary-flat.pdf

Life sciences study design

All studies must disclose on these points even when the disclosure is negative.

Sample size	Sample size were not predetermined using statistical methods. Cryo-EM sample size are determined by the availability of microscope time and the number and quality of particles to obtain the reported structures. The sample size of each dataset is indicated in Table and the image-processing procedures. For cellular assays, the sample size was chosen on the basis of commonly used sample sizes in the field.
Data exclusions	Cryo-EM images were excluded based on CTF max resolution parameters. Particles in 3D classification in RELION software with poor structural features were excluded. These procedures are general practice in the field of cry-EM single particle analysis.
Replication	Cell-based experiments were repeated at least three times in independent experiments. All attempts at replication were successful.
Randomization	Randomization was performed when calculating Fourier-shell correlation of half maps in the cryo-EM analyses.
Blinding	Blinding was not performed as subjective analysis was not needed and no group allocation was performed for this study.

Reporting for specific materials, systems and methods

We require information from authors about some types of materials, experimental systems and methods used in many studies. Here, indicate whether each material, system or method listed is relevant to your study. If you are not sure if a list item applies to your research, read the appropriate section before selecting a response.

Materials & experimental systems

n/a	Involved in the study	
	x	Antibodies
	x	Eukaryotic cell lines
x		Palaeontology and archaeology
x		Animals and other organisms
x		Clinical data
x		Dual use research of concern
x		Plants

Methods

n/a	Involved in the study	
x		ChIP-seq
x		Flow cytometry
x		MRI-based neuroimaging

Antibodies

Antibodies used	Primary antibodies: Anti-DDDDK-tag mAb-HRP-DirecT (anti-FLAG, 1:2500 dilution; Medical & Biological Laboratories, M185-7), anti-Myc (1:2500 dilution; Medical & Biological Laboratories, 192-3), anti-Strep II (1:2000 dilution; Medical & Biological Laboratories,

	M211-3), anti-HA tag (1:2500 dilution; Medical & Biological Laboratories, M180-3), and anti-β-actin (1:2500 dilution; Santa Cruz Biotechnology, sc-47778, Lot no. J0421); secondary antibodies: rabbit anti-mouse IgG H&L (HRP) (1:2500 dilution; Abcam, ab6728)
Validation	These antibodies were validated by manufacturers and the related information can be found at the company websites.

Eukaryotic cell lines

Policy information about cell lines and Sex and Gender in Research

Cell line source(s)	ExpiSf9 and Expi293F cells were purchased from ThermoFisher Scientific (cat# A35243 and A14635).<br>HEK293T cells were purchased from the American Type Culture Collection (ATCC, ATCC CRL-3216).
Authentication	The cell lines were authentified by the manufacturers and no further authentification was performed.
Mycoplasma contamination	Not tested.
Commonly misidentified lines<br>(See ICLAC register)	No commonly misidentified cells were used in this study.

Plants

Seed stocks	N/A
Novel plant genotypes	N/A
Authentication	N/A
